# In-depth *in vitro* Evaluation of the Activity and Mechanisms of Action of Organic Acids and Essential Oils Against Swine Enteropathogenic Bacteria

**DOI:** 10.3389/fvets.2020.572947

**Published:** 2020-11-10

**Authors:** Manuel Gómez-García, Héctor Argüello, Héctor Puente, Óscar Mencía-Ares, Sandra González, Rubén Miranda, Pedro Rubio, Ana Carvajal

**Affiliations:** Department of Animal Health, Faculty of Veterinary Medicine, Universidad de León, León, Spain

**Keywords:** swine, organic acids, single essential oil compounds, feed additive, bacterial viability, antimicrobial mechanism

## Abstract

Alternative antimicrobials require a deep understanding of their action mechanisms by *in vitro* assays which support science-based field use. This study focuses on the characterization of bactericidal mechanisms of potential antimicrobial compounds, two organic acids and three single essential oil (EO) compounds against swine enteropathogenic bacteria *Escherichia coli, Salmonella enterica* subsp. *enterica* serovar Typhimurium, and *Clostridium perfringens*. Target concentrations of the compounds were evaluated using the inhibitory potential of the vapor phase and bacterial viability after short-term exposure, while cell targets were disclosed using flow cytometry (FC), Fourier-transform infrared (FTIR) spectroscopy, and scanning electron microscopy (SEM). All tested compounds exhibited vapor phase activity against the three bacterial species, except sodium salt of coconut fatty acid distillates against *C. perfringens*. Survival test results evidenced that effects on bacterial viability were concentration dependent and higher in single EO compounds than in organic acids. In detail, thymol and its isomer carvacrol were the most effective compounds. Further characterization of thymol and cinnamaldehyde activity revealed that thymol main target was the cell membrane, since it caused striking damages in the membrane permeability, integrity and composition evidenced by FC and FTIR in the three enteric pathogens. In contrast, cinnamaldehyde was more effective against enterobacteria than against *C. perfringens* and only caused slightly damages at the highest concentration tested. Its target at the molecular level differed between enterobacteria and *C. perfringens* isolates. The SEM micrographs allowed us to confirm the results previously obtained for both EO compounds by other techniques. Altogether, the study showed the straight effect of these antimicrobials, which could constitute relevant information to optimize their feed inclusion rates in field studies or field use.

## Introduction

The emergence of antimicrobial resistance (AMR) is a major worldwide public health concern, which reduces the efficacy of antibiotic treatments in human and animal infections (1). Current policies aim at reducing the use of antibiotics (ABs) to mitigate the selective pressure on AMR rising (2). In this way, searching for alternative strategies which replace or mitigate the use of ABs to treat infectious diseases is of vital importance (3, 4). In animal medicine, in particular livestock medicine, potential antimicrobial compounds such as organic acids and plants extracts have been tested as feed additives, evaluating their potential impact on growth and health (4–6).

These feed additives could be of particular interest in the control of enteric infectious disorders in swine (7–10). Their efficient use goes through the estimation of the concentrations at which they are effective by *in vitro* activity assays (11) and the strategic administration to animals by *in vivo* experiments (7, 8, 12) which evaluate the efficacy of these compounds against pathogenic bacteria. Disclosing the mechanisms of action of these compounds is of relevance for an efficient use against pathogens. Thus, numerous studies have provided information about cell targets and the modes of action of organic acids and essential oils (3, 13–15). Despite the knowledge acquired, these studies stress that the mode of action and target structures or processes in pathogenic bacteria are not completely understood (3, 13–15). In this context, information regarding their effect on bacterial viability after short-term exposure and the inhibitory potential of vapor phase of these compounds has been poorly revised (13, 16). It also draws attention the usefulness of precise techniques such as flow cytometry (FC) or Fourier-transform infrared (FTIR) spectroscopy to add information about the mode of action of single alternative compounds. Indeed, both techniques are considered as powerful non-destructive tools to obtain physiological, compositional, and structural information of bacteria after the exposure of different experimental conditions or compounds (17, 18).

Searching for efficient alternatives to antimicrobials, it is essential to perform combined studies where the mode of action analyses are targeted at efficient concentrations and using field isolates of enteropathogenic bacteria. Altogether, this sort of study could provide relevant information when optimizing their feed inclusion rates and antimicrobial activities of EOs constituents (15, 16), supporting with scientific data their application in animal production (3).

This study aims at extending the knowledge acquired in the *in vitro* evaluation of the antimicrobial activity of two organic acids, formic acid and sodium salt of coconut fatty acid distillates, three single essential oil (EO) compounds, thymol, cinnamaldehyde, and carvacrol against a selection of animal enteric pathogenic bacteria (11). Here we have introduced results of their effects on the bacterial viability after short-term exposure or the inhibitory potential of their vapor phase. The mechanism of action of thymol and cinnamaldehyde was also studied by means of their effects on the permeability, structure, composition and morphology in the bacteria tested, with special focus on the cell membrane.

## Materials and Methods

### Bacterial Isolates and Compounds Tested

All experiments were performed using five isolates of *Escherichia coli, Salmonella enterica* subsp. *enterica* serovar Typhimurium (onwards *S*. Typhimurium), and *Clostridium perfringens* (type A, alpha toxin) from pig origin and one strain (*S*. Typhimurium CECT 443) from the Spanish Type Culture Collection (Table 1). Except CECT 443, all isolates belong to the collection of the Animal Health Department and were isolated from fecal samples from diarrhea outbreaks on Spanish pigs. Their characteristics and growing conditions are detailed elsewhere (11).

**Table 1:** Bacterial isolates used in the study and concentration range in part per million (ppm) evaluated for each tested compound.

	**Bacterial species**	***Escherichia coli***		***Salmonella enterica*** **subsp**. ***enterica*** **serovar Typhimurium**		***Clostridium perfringens***	
	**Isolates**	**EC 61**	EC 67^*^	SP11^*^	**CECT 443**	**CP 34**	CP 52^*^
Formic acid	½MBC_low_	1,200 ppm		1,200 ppm		600 ppm	
	MBC_low_	2,400 ppm		2,400 ppm		1,200 ppm	
	MBC_high_					2,400 ppm	
	2MBC_high_	4,800 ppm		4,800 ppm		4,800 ppm	
Sodium salt of coconut fatty acid distillates	½MBC_low_	-		-		8 ppm	
	MBC_low_					16 ppm	
	MBC_high_					32 ppm	
	2MBC_high_					64 ppm	
Cinnamaldehyde	½MBC_low_	600 ppm		600 ppm		150 ppm	
	MBC_low_	1,200 ppm		1,200 ppm		300 ppm	
	MBC_high_	2,400 ppm		2,400 ppm		600 ppm	
	2MBC_high_	4,800 ppm		4,800 ppm		1,200 ppm	
Thymol	½MBC_low_	600 ppm		300 ppm		600 ppm	
	MBC_low_	1,200 ppm		600 ppm		1,200 ppm	
	MBC_high_			1,200 ppm		
	2MBC_high_	2,400 ppm		2,400 ppm		2,400 ppm	
Carvacrol	½MBC_low_	150 ppm		150 ppm		150 ppm	
	MBC_low_	300 ppm		300 ppm		300 ppm	
	MBC_high_	600 ppm		600 ppm		600 ppm	
	2MBC_high_	1,200 ppm		1,200 ppm		1,200 ppm	

*Minimum bactericidal concentration (MBC) values tested: twice the highest MBC (2MBC_high_), the highest MBC (MBC_high_), the lowest MBC (MBC_low_), and half the lowest MBC (½MBC_low_) previously obtained for each bacterial species. When MBC_low_ and MBC_high_ were the same, only three concentrations were tested*.

* *Antibiotic multi-resistant isolate*.

In accordance with previous authors (18, 19), the assays were carried out with swine farm isolates with the aim of providing relevant information which can be extrapolated to field conditions and in *Salmonella* offers a particular comparison between *S*. Typhimurium field isolate and a collection strain (*S*. Typhimurium CECT 443). For each bacterial specie, one antibiotic multi-resistant isolate was also selected to investigate if the antibiotic multi-resistant profile has influence on the susceptibility to compounds.

Two organic acids, formic acid (purity 85%) and sodium salt of coconut fatty acid distillates (67%), three EO compounds, cinnamaldehyde (97–98%), thymol (99%), and carvacrol (99%), were included in this study. All the tested compounds were provided by Norel SA (Madrid, Spain). Sodium salt of coconut fatty acid distillates and thymol, which were obtained as powder compounds, were diluted in distilled sterile water at 10,000 ppm (w/v) and in sterile propylene glycol (1:1) (Sigma-Aldrich), respectively.

### Effect of the Vapor Phase

The effect of the vapor phase of each pure tested compound was determined using the inverted Petri dish method as previously described (20).

In brief, 100 μl of a bacterial suspension with a turbidity of 0.5 on the McFarland scale (Sensititre nephelometeter, Trek Diagnostic Systems), equivalent to 1,5 × 10^8^ colony-forming units (CFU)/ml, were distributed onto tryptic soy agar (TSA) (Scharlab) plates for *E. coli* and *S*. Typhimurium or fastidious anaerobe agar (FAA) (Neogen) plates for *C. perfringens*. Sterile disks of 6 mm were impregnated with 15 μl of each tested compound and placed in the lid of the plates. Disks impregnated with propylene glycol were included to check the lack of vapor phase activity. Plates were incubated at 37°C in aerobic and anaerobic conditions, respectively, for TSA and FAA and each assay was performed by triplicate.

### Survival Test

The concentrations used in this study were selected according to the minimum bactericidal concentration (MBC) values obtained in a previous research (11). The concentrations evaluated were twice the highest MBC (2MBC_high_), the highest MBC (MBC_high_), the lowest MBC (MBC_low_), and half the lowest MBC (½MBC_low_) obtained for each bacterial species. If MBC_high_ and MBC_low_ overlapped, the concentrations tested were three (Table 1). Like thymol in the previous assay and in accordance with the manufacturer's instructions, the rest of EOs were diluted 1:1 in sterile propylene glycol in order to facilitate their dilution in each of the broths used in this study, Mueller-Hinton (Cultimed) and brain heart infusion (BHI) (Merck), previously adjusted to pH 6.0 using 50 mM sodium phosphate buffer, minimizing the effect of the pH on the evaluation of antimicrobial activity. The sodium salt of coconut fatty acid distillates was only evaluated against *C. perfringens* owing to its lack of activity against *E. coli* and *S*. Typhimurium in the range of concentrations tested (Table 1).

Survival test was performed as described elsewhere (18, 19), but without sample centrifugation before exposing the bacteria to the tested compounds. Prior to each assay, 3–4 fresh bacterial colonies (after 24–36 h incubation) were suspended in fresh broth and grown under optimal conditions to stationary phase. Next, 950 μl of the bacterial culture were mixed with 50 μl of specific broth plus each compound concentrated 20 times to the final concentration tested. Controls were grown in the same broth. Samples were incubated for 90 min at room temperature in complete darkness. The protocol used for the inoculum preparation and the exposition of culture to sublethal and lethal concentrations of products was common for the subsequent experiments.

After the exposition, 10-fold series dilutions were carried in phosphate buffered saline (PBS) and aliquots of 100 μl were plated in duplicate onto solid media, TSA for *E. coli* and *S*. Typhimurium while FAA was used for *C. perfringens*. Plates were incubated under optimal conditions, reductions in viable bacteria were estimated by calculating the number of CFUs in each broth. Three replicates were performed for each exposure using three different fresh cultures.

### Membrane Integrity Test Using Flow Cytometry (FC)

The effect of thymol and cinnamaldehyde on membrane permeability was evaluated by propidium iodide or PI (Invitrogen) uptake as previously described (18) avoiding centrifugation. After 90 min exposure, bacterial suspensions with and without product incubated for 90 min were diluted 1:10 in phosphate-buffered saline (PBS, pH 7.4) and PI was then added to a final concentration of 0.1% (v/v). After 10 min incubation at room temperature in complete darkness, samples were measured using a CyAn-adp flow cytometer (Beckman Coulter). The configuration of the instrument was forward scatter (FS), side scatter (SS) and red fluorescence (613/20 nm) for PI with a laser excitation of 488 nm. Data obtained were analyzed using Summit version 3.1 software (Cytomation). Membrane integrity experiments were also performed in triplicate.

### Changes in the Medium Infrared (MIR) Spectra

FTIR analysis was used to study the effect of thymol and cinnamaldehyde after 90 min exposure using a previously reported methodology with some modifications (18, 19). Infrared spectra were recorded with a FTIR spectroscope (JASCO 4700 FTIR) over the wavelength range of 4.000–700 cm^−1^ with an interval of 1 cm^−1^ and a spectral resolution of 4 cm^−1^. The final spectrum of each sample was achieved averaging 20 scans and transformed with Spectra Manager Version 2.0 developed by JASCO Corporation. This transformation included normalization, 0 setting absorption at 1.800 cm^−1^ and setting at maximal absorption around 1.650 cm^−1^, smoothing and second derivative using Savitzky-Golay algorithm. According to Alvarez-Ordóñez and Prieto (21), five informative spectral windows assigned to particular functional groups were selected within the MIR spectrum: w_1_ window (3,000–2,800 cm^−1^) or membrane fatty acids and amino acids region, w_2_ (1,800–1,500 cm^−1^) or protein, lipids and nucleic acids region, w_3_ (1,500–1,200 cm^−1^) with no defined target, w_4_ (1,200–900 cm^−1^) or polysaccharide region, and w_5_ (900–700 cm^−1^) or fingerprint region.

Spectral data were recorded in ASCII format and the averages of three independent replicates of each exposure and control were graphically represented using Excel 2010 (Microsoft). Finally, Metaboanalyst 4.0 software was used to identify the spectral region with major differences between the spectra of the exposures to 2MBC_high_ and those of the control using Random forest (RF) as well as to analyze and sort out the absorbance spectra using principal component analysis (PCA).

### Scanning Electron Microscopy (SEM)

One isolate of each bacterial species tested (*E. coli* EC 67, *S*. Typhimurium CECT 443 and *C. perfringens* CP 34) was used to study the effect of the exposure to different concentrations of thymol and cinnamaldehyde on bacterial morphology. After 90 min exposure at room temperature, treated bacterial cultures and controls were prepared following a previously described method (18, 22). It included a fixation with 2.5% (v/v) glutaraldehyde followed by a dehydration with graded ethanol series and a drying with the critical point method. The samples were mounted on aluminum stubs with conducting carbon ribbon and coated by sputter coater (SCD 004, Balzers). Digital images were taken using a scanning electron microscope (JEOL 6480LW) at a 20 kV accelerating voltage.

### Statistical Analysis

Effect of the vapor phase, survival test and membrane integrity using FC assay data were tested for normality by Kolmogorov-Smirnov test and statistical differences were evaluated using either ANOVA or Kruskal-Wallis tests. The nominal *p*-value for statistical significance was *p* < 0.05. The similarity between FTIR spectra of the three replicates of each exposure and isolate was also measured using individual Pearson's product-moment correlation coefficient. All the analyses were carried out with SPSS Statistics version 24 (IBM).

## Results

### Effect of the Vapor Phase

The measures of inhibition zone diameter (mm) obtained for each tested compound and bacterial species tested are given in Table 2. Sodium salt of coconut fatty acid distillates did not show any activity. Carvacrol as well as formic acid exhibited the highest volatile activity against the three tested species whose measures differed significantly (*p* < 0.05) with respect to those obtained for the rest of products in most of cases (Table 2). In addition, *C. perfringens* was the most susceptible bacterial species to volatile components. The intra-species susceptibility to each compound was relatively homogeneous, except for cinnamaldehyde against the three bacterial species and thymol and carvacrol against *S*. Typhimurium.

**Table 2:** Mean of the three measures of inhibition zone diameters (mm) and standard deviation obtained for the disks impregnated with each tested compound against *E. coli* (EC 61 and EC 67), *S*. Typhimurium (SP 11 and CECT 443), and *C. perfringens* (CP 34 and CP52) isolates.

	**Formic acid**		**Sodium salt of coconut fatty acid distillates**		**Cinnamaldehyde**		**Thymol**		**Carvacrol**	
**Isolate**	**Mean**	**SD**	**Mean**	**SD**	**Mean**	**SD**	**Mean**	**SD**	**Mean**	**SD**
EC 61	36.00^*^	1.73	0.00	0.00	33.00	2.65	28.00	3.46	42.50^*^	3.54
EC 67	37.67^*^	0.58	0.00	0.00	24.33	1.53	26.67	3.06	38.00^*^	3.46
SP 11	35.33^*^	0.58	0.00	0.00	23.67	3.21	26.67	5.77	43.33^*^	3.06
CECT 443	39.33	1.15	0.00	0.00	39.00	1.00	48.00^*^	2.00	60.67^*^	1.15
CP 34	48.00^*^	2.00	0.00	0.00	36.67	5.03	28.33	3.21	44.00^*^	3.46
CP 52	55.33^*^	4.16	0.00	0.00	44.67	2.31	30.00	2.00	48.33^*^	0.58

* *Denotes the means (mm) which showed statistically significant differences compared with the rest (p < 0.05)*.

### Survival Test

**Figure 1:**
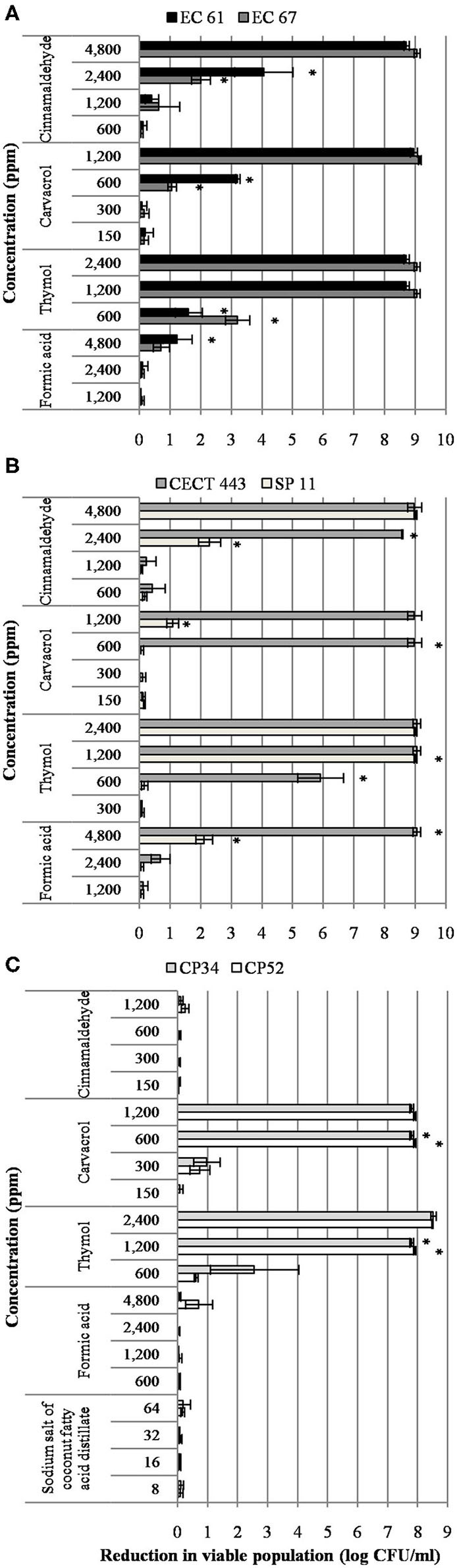
Reduction in viable colonies (log CFU/ml) of *E. coli* **(A)**, *S*. Typhimurium **(B)**, and *C. perfringens* **(C)** after the exposure to different concentrations of the tested compound for 90 min at room temperature. Data are expressed as mean of the three independent replicates ± standard deviations. *Denotes the lowest concentration of product in which population viability differed significantly with respect to controls (*p* < 0.05).

A concentration dependent reduction in bacterial viable populations was observed after short-term exposure to most of the tested compounds for at least one of the three bacterial species (Figure 1). It is worth noting that initial concentration of culture was approximately 8 and 9 log of CFU/ml for *C. perfringens* and enterobacteria, respectively. In general, viability was more affected by the tested EO compounds than by the organic acids. Interestingly enough, no effect of sodium salt of coconut fatty acid distillates, formic acid and cinnamaldehyde was observed against *C. perfringens* isolates. Formic acid showed also low activity against *E. coli* and *S*. Typhimurium. However, thymol was the most active compound and significant reductions (*p* < 0.05) of 8.9, 9.0, and 7.8 log of CFU/ml for *E. coli, S*. Typhimurium and C. *perfringens*, respectively, were achieved at 1,200 ppm.

In contrast to other bacterial species, *S*. Typhimurium showed isolate variability in the susceptibility to the different concentrations tested. Indeed, the strain *S*. Typhimurium CECT 443 was more sensitive than *S*. Typhimurium SP 11.

The survival test allowed us to select two compounds, thymol, and cinnamaldehyde, which were further researched to determine their mechanism of action.

### Membrane Integrity Test Using Flow Cytometry

The assessment of PI uptake owing to loss in the membrane integrity after exposure to different concentrations of thymol and cinnamaldehyde for 90 min was performed using FC. When considering the results obtained in section Survival Test, we only tested two concentrations, MBC_high_ (600 ppm) and 2MBC_high_ (1,200 ppm), of cinnamaldehyde against *C. perfringens* isolates.

**Figure 2:**
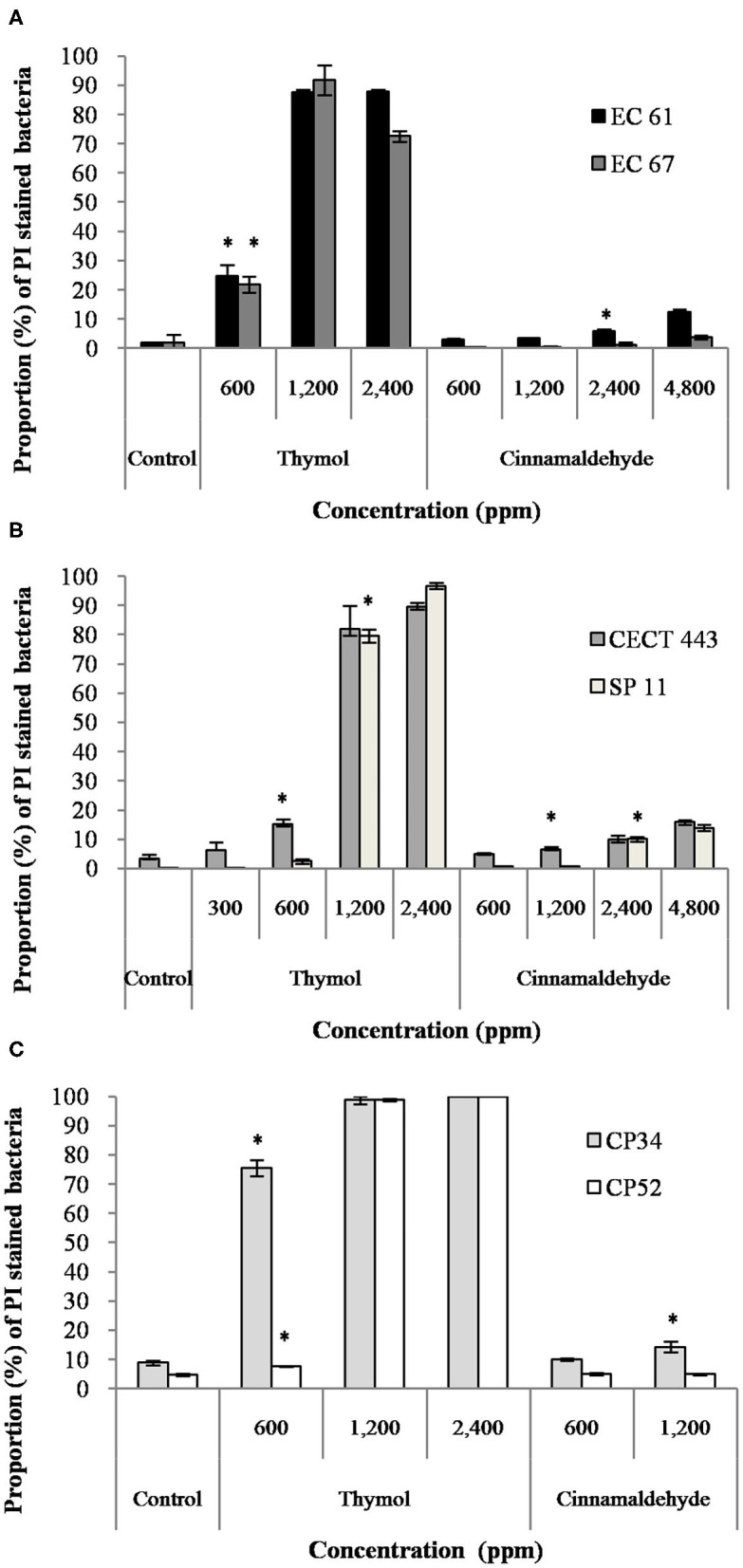
Proportion of propidium iodide (PI) stained bacteria of *E. coli* **(A)**, *S*. Typhimurium **(B)**, and *C. perfringens* **(C)** among controls without products and after the exposure to different concentrations of thymol and cinnamaldehyde for 90 min at room temperature. Data are expressed as mean of the three independent replicates ± standard deviations. *Denotes the lowest concentration of product in which PI stained bacteria significantly differed to controls (*p* < 0.05).

The mean percentage of bacteria stained with PI after the exposure to both tested compounds is given in Figure 2. Both EOs caused concentration-dependent changes in the membrane integrity for the three bacterial species, although alterations in the membrane were remarkably higher for thymol with values of almost 100% of PI stained bacteria and statistically significant differences (*p* < 0.05) compared to the control, even with exposures to ½MBC_low_ or MBC_low_ (600 ppm) in most of isolates. However, cinnamaldehyde activity on membrane integrity was variable, with low activity against *E. coli* and *C. perfringens*. Indeed, significant differences (*p* < 0.05) between exposed cultures and their controls were only observed for *E. coli* EC 61 and *C. perfringens* CP 34 after exposure to MBC_high_ (2,400 ppm) and 2MBC_high_ (1,200 ppm) of cinnamaldehyde, respectively.

### Changes in the Medium Infrared (MIR) Spectra

We were able to measure the effects of the different concentrations of thymol and cinnamaldehyde on cell structure and composition through FTIR spectra. From the results obtained in the previous two sections, only the highest concentration of cinnamaldehyde (2MBC_high_, 1,200 ppm) was evaluated against *C. perfringens*. The RF analysis allowed for the identification of the most variable wavelengths for each EO and bacterial species. Thymol caused changes in the region w_4_ (polysaccharide) for the three bacterial species while cinnamaldehyde in the w_4_ for *E. coli* and *S*. Typhimurium and in the w_3_ region (not defined) for *C. perfringens* isolates. We observed high reproducibility among replicates with an average Pearson's coefficient ρ > 0.95.

**Figure 3:**
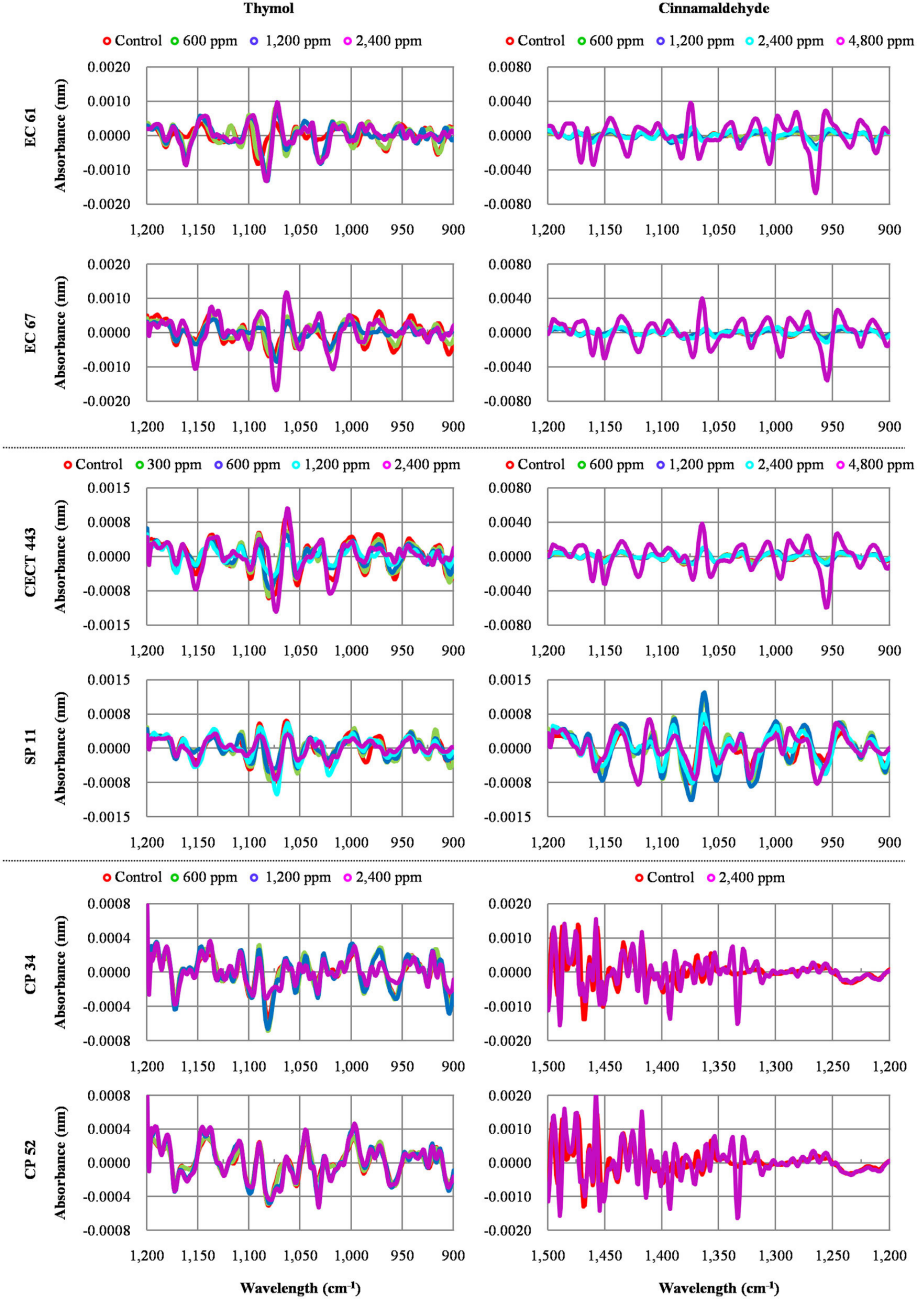
Average values of Fourier Transform Infrared (FTIR) absorbance spectra of the three replicates of the selected windows obtained for each isolate tested after the exposure to different concentrations of thymol and cinnamaldehyde (green line, ½MBC_low_; blue line, MBC_low_; light blue line, MBC_high_; purple line, 2MBC_high_) and without product (red line, control) for 90 min at room temperature.

**Figure 4:**
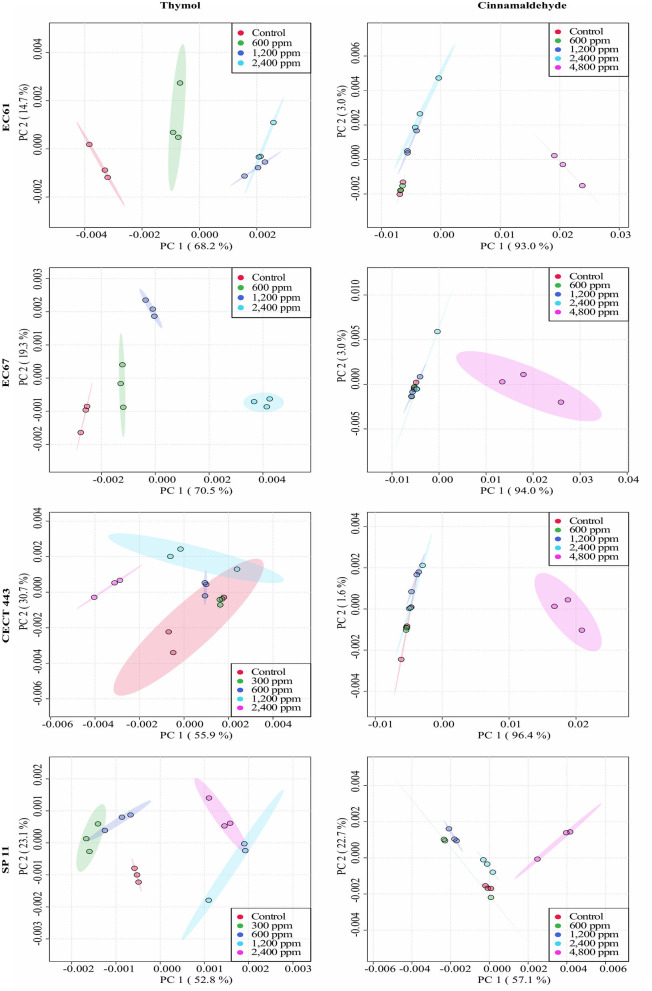
Principal component analysis (PCA) from ASCII data of the FTIR absorbance spectra of the selected windows obtained for each isolate tested of *E. coli* and *S*. Typhimurium after the exposure to different concentrations of thymol and cinnamaldehyde (green shaded areas and points, ½MBC_low_; blue shaded areas and points, MBC_low_; light blue shaded areas and points, MBC_high_; purple shaded areas and points, 2MBC_high_) and without compound (red shaded areas and points, control) for 90 min at room temperature.

**Figure 5:**
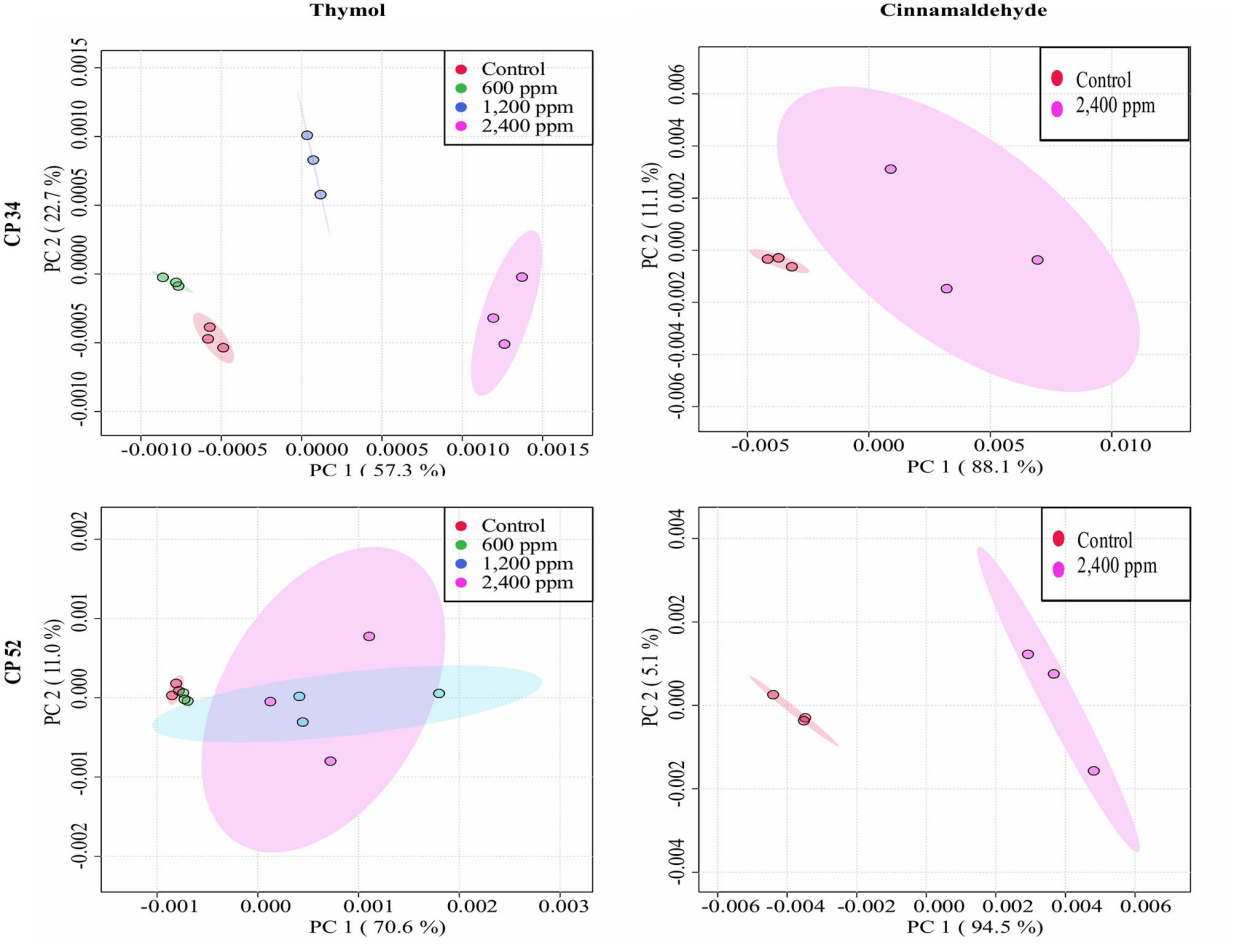
Principal component analysis (PCA) from ASCII data of the FTIR absorbance spectra of the selected windows obtained for each isolate tested of *C. perfringens* after the exposure to different concentrations of thymol and cinnamaldehyde (green shaded areas and points, ½MBC_low_; blue shaded areas and points, MBC_low_; light blue shaded areas and points, MBC_high_; purple shaded areas and points, 2MBC_high_) and without compound (red shaded areas and points, control) for 90 min at room temperature.

The average of processed FTIR spectra of the three replicates of the selected windows obtained for each isolate showed differences between treated bacteria and the respective controls (Figure 3). The changes observed were concentration dependent, except for thymol against *C. perfringens* CP 52 isolate. The PCAs from ASCII data of selected windows emphasized that major spectral modifications occurred after the exposure to the highest concentration tested for both tested compounds. The segregation of 2MBC_high_ spectra was less evident for samples exposed to thymol than for those exposed to cinnamaldehyde (Figures 4, 5).

### Scanning Electron Microscopy (SEM)

**Figure 6:**
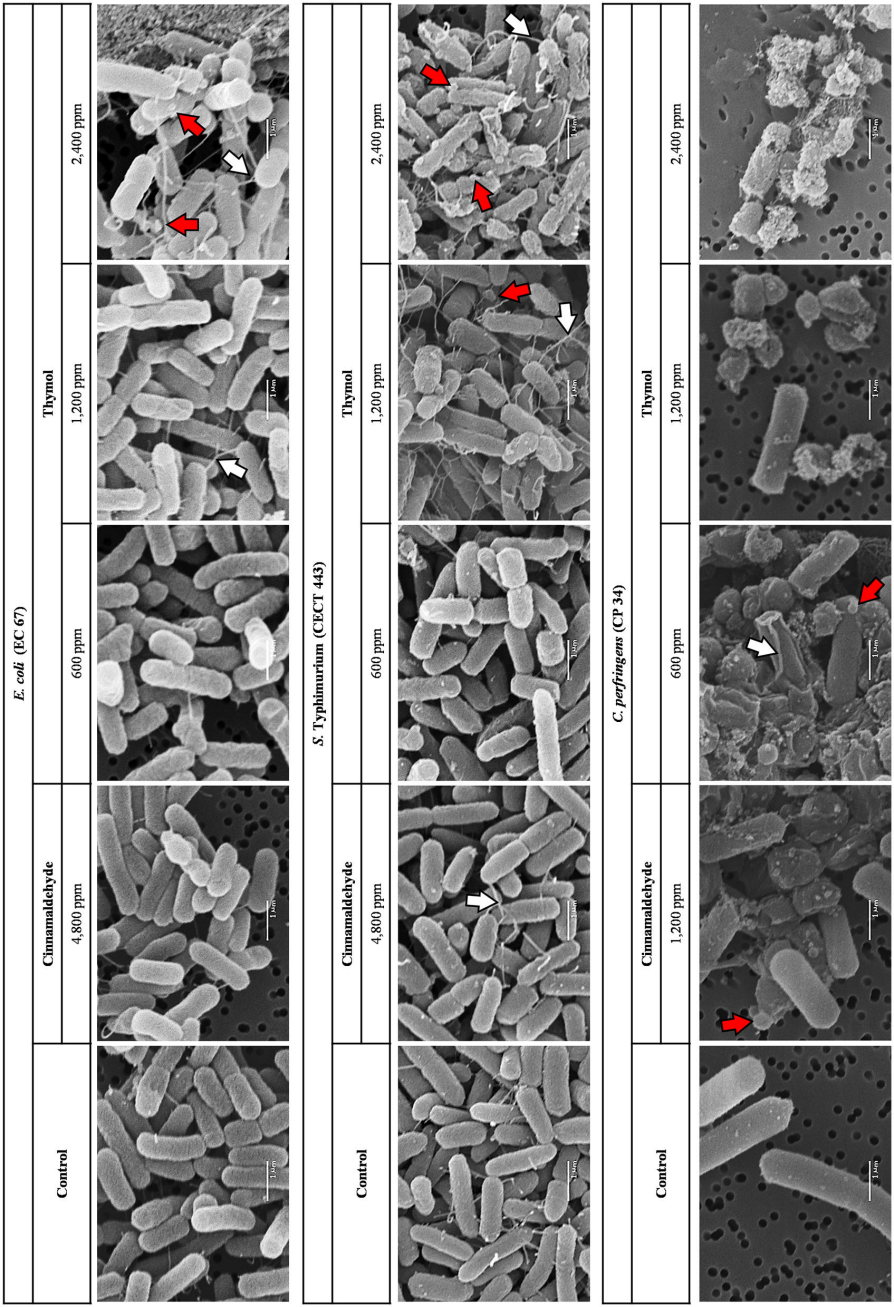
SEM micrographs of the selected isolates of *E. coli, S*. Typhimurium, and *C. perfringens* exposed at the three highest concentrations tested of thymol and to 2MBC_high_ of cinnamaldehyde for 90 min at room temperature. Depending on the color inside the arrows, the red arrows point to blends and microvesicles while the white arrows show networks of intercellular and extending nanotubes.

Taking into account the results obtained in previous assays, thymol was tested at the three highest concentrations established for each bacterial species using the SEM technique, while cinnamaldehyde was only tested at the highest concentration or 2MBC_high_ (Figure 6).

Concentration-dependent effects were observed after thymol exposure in the three bacterial species studied. Blebs, microvesicles, and networks of intercellular and extending nanotubes were the most common effects. Although nanotubes were observed in the control without compound for *S*. Typhimurium CECT 443, they increased with the concentration of thymol, being particularly evident at 2MBC_high_ (2,400 ppm) when the cells also showed a loss in their structural integrity including deformations. Finally, remarkable damage on the surface was even detected after the exposure to the lowest concentration tested (½MBC_low_, 600 ppm) in *C. perfringens* CP 34, with a high degree of cell lysis and collapse.

In contrast, SEM allowed us to confirm the lack of apparent morphological and physical changes in *E. coli* or *S*. Typhimurium cells after the exposure to cinnamaldehyde. Blebs and microvesicles as well as certain loss of structural integrity were observed in *C. perfringens* cells after exposure to 2MBC_high_ of cinnamaldehyde.

## Discussion

In a previous study, we added information to the existing knowledge on the usefulness of organic acids and essential oils against enteropathogenic bacteria, focusing on three relevant pathogens in swine (11). The information gathered was used to evaluate, at target concentrations and through different acknowledged techniques (18), the mechanisms by which these compounds exert their activity against *E. coli, S*. Typhimurium, and *C. perfringens*. It is noteworthy that *E. coli* and *C. perfringens* showed a homogeneous intra-species response to these compounds while *S*. Typhimurium sensitivity to vapor phases was higher in the collection strain *S*. Typhimurium CECT 443 than in the field multi-resistant isolate *S*. Typhimurium SP 11, which highlights the relevance of using field isolates to achieve maximum results reliability.

Using the survival test, we evaluated the effect of each tested compound on bacterial viability after short-term exposure. We tested and compared a range of concentrations, including the highest and lowest MBC values for each bacterial species, information gathered in a previous research (11). The results evidenced that short term activity of formic acid and sodium salt of coconut fatty acid distillates was lower compared to the EO compounds in the three bacterial species at the concentrations evaluated. While these two organic acids did not achieve more than two log reductions after 90 min exposure, an average reduction of 7.37 log in viable populations was obtained with the highest concentration of the EO compounds tested (2MBC_high_), cinnamaldehyde against *C. perfringens* was the only exception. These findings show that despite the fact that MBC of organic acids are relatively low (11), they may require longer exposure than EO compounds to exert their direct antimicrobial effect. Interestingly enough, we observed a higher bacteria viability reduction using the single EO compounds tested compared to previous studies, which under similar conditions and targeting the same bacterial species, analyzed other natural feed additives (18, 19). It is remarkable, that our methodology avoided sample centrifugation before the exposure to compounds in order to prevent any damage in cell membrane according to de Nova et al. (18), so the EO compounds activity was not influenced by the sample manipulation. Moreover, our results highlight the differences in compound efficacy against the two enterobacteria and *C. perfringens*. This latter required higher concentrations to achieve similar reductions or was not susceptible at the time of exposure, i.e., cinnamaldehyde. As well, differences between thymol and its isomer carvacrol with a very similar pattern of activity and action mechanism (23, 24) were also evidenced. While thymol exhibited high antimicrobial activity within the exposure time, as previously described (25), carvacrol had lower efficacy at the same concentrations, a result that may be attributed to technical aspects of the manufacturing and storage processes or biological factors related to their source plant (26). However, the differences in the chemical configuration of hydroxyl group could also determine the differences observed (27).

The antibacterial activity of these compounds may be partially achieved through their vapor phase (28). Except for sodium salt of coconut fatty acid distillates, the volatile components of the compounds inhibited the growth of the three bacterial species tested. The lack of volatile activity of this salt could partially be explained by the low proportion of its active volatile compounds at the concentration evaluated. The narrow inhibition zone observed in thymol could also be a consequence of its dilution 1:1 in propylene glycol since previous studies have shown the vapor activity of thyme EO, whose major component is thymol, against *Streptococcus suis* (20). Nevertheless, discrepancies between both results can also be explained by the fact of presence of minor compounds in the thyme EO composition which appear to enhance the activity of thymol as previously described (29). Finally, the higher susceptibility of *C. perfringens* to vapor phases with regard to the two enterobacteria tested could be related to the differences in their complex cell wall as previously described (30).

Further experiments were focused on two EOs with different hypothetical mechanism of action, thymol and cinnamaldehyde (23, 31, 32). Both EOs revealed striking short-term and vapor phase activities but showed differences in the activity against *C. perfringens*. Carvacrol was not further evaluated as its activity was similar to thymol and its potential mechanism of action may be the same as its phenolic isomer thymol (33). Thus, through three different techniques, we aimed at deciphering targets and mechanisms of action of each compound.

Assays with PI revealed that exposure to effective concentrations of thymol caused striking changes in the membrane permeability, similarly to previous studies in *Salmonella, Streptococcus* and *Staphylococcus* (25, 34). This result was confirmed by the analysis of FTIR spectra as the exposure to thymol modified the w_4_ spectral region in the three bacterial species. The w_4_ window provides information on alterations in carbohydrates and polysaccharides of the cell wall due to C-O-C and C-O-P stretching as well as in nucleic acids due to P=O symmetric stretching (21). Results in both experiments were concentration dependent; PI intake increased by thymol concentration and more prominent FTIR spectral changes were observed at the highest concentration studied.

Cinnamaldehyde, in contrast, showed limited activity on the membrane permeability according to the low PI intake particularly for *C. perfringens* after exposure to high bactericidal concentrations. The analyses of FTIR spectra expanded this information. Indeed, some activity in the w_4_ region, linked to membrane damage was observed for *E. coli* and *S*. Typhimurium, but not for *C. perfringens* which showed changes in w_3_ region, linked to proteins, fatty acids and compounds with phosphate groups which are mainly due to C-O symmetric stretching (21). Lipids and proteins of bacterial cell membranes could be the main targets of cinnamaldehyde although variations are expected depending on the structure and composition of the cell wall which differs between Gram-negative and Gram-positive bacteria (23, 25, 34). Finally, in contrast to thymol, cinnamaldehyde did not modified FTIR spectrum after the exposure of ½MBC_low_, MBC_low_, or even MBC_high_ in neither of both enterobacteria when compared to controls. Differences were only observed after exposure to 2MBC_high_, supporting the survival test results in which only the 2MBC_high_ concentration was able to achieve near to 100% reductions in bacterial viability.

Regarding the analysis of FTIR spectra, some authors have reported that the spectral information obtained by FTIR consistently provided structural information of biological molecules (17). In our case, both windows selected, w_4_ for thymol against all the bacterial species and cinnamaldehyde against enterobacteria as well as w_3_ for cinnamaldehyde against *C. perfringens*, have been identified as the most reproducible and variable to evaluate the mechanism of action of other alternative products to antibiotics in similar studies (18, 19) However, changes in the w_1_ spectral region, associated with membrane fatty acids, have also been identified after the exposure to bacteriocins by others authors (35, 36). These changes are related to the dissipation of proton motive force and leakage of intracellular contents which have also been associated to the mechanism of action of phenolic isomers (33).

By SEM we confirmed the results pointed out by the previous techniques. Effects on the surface of the bacteria such as fissures, blebs, microvesicles and nanotubes were evident after exposure to thymol. These changes occur as part of the membrane damage (18) and were already reported in *S*. Typhimurium after thymol exposure in SEM micrographs with low sharpness (25). By avoiding any centrifugation in the methodology, we could verify that the changes observed were only owing to the effect of the compound. This fact may also explain the minor effect observed after the short-term exposure to cinnamaldehyde on *E. coli* and *S*. Typhimurium as opposed to previous studies (37). In addition, alterations observed on the surface of *C. perfringens* CP 34 after the exposure to this tested compound could explain the significant differences between the results of PI uptake obtained at the same concentration (2MBC_high_) and the control detected in the membrane integrity test. Although the effect of cinnamaldehyde may be time dependent, our result evidencing limited bacterial membrane damage after cinnamaldehyde exposure, go in line with previous studies (23, 38).

This study shows the direct activity of different single EO compounds, principally thymol and cinnamaldehyde, and to a lower extent formic acid, after short-term exposure against *E. coli, S*. Typhimurium and *C. perfringens*. The activity was concentration dependent and partially associated to the vapor phase of these compounds. Thymol activity was associated to alterations in the bacteria cell wall, while cinnamaldehyde activity was associated to the cell wall for both enterobacteria and proteins and fatty acids changes for *C. perfringens*. Altogether, the study demonstrates the direct effect of these antimicrobials against enteric pathogens and their potential usefulness at the concentrations evaluated.

## Data Availability Statement

The raw data supporting the conclusions of this article will be made available by the authors, without undue reservation.

## Author Contributions

MG-G, HA, PR, and AC conceived and designed the experiments, analyzed the data, and wrote and revised the manuscript. MG-G, HP, ÓM-A, SG, and RM performed the experiments. All authors contributed to the article and approved the submitted version.

## Conflict of Interest

The authors declare that the research was conducted in the absence of any commercial or financial relationships that could be construed as a potential conflict of interest.
